# Anti-atherogenic potential of jujube, saffron and barberry: anti-diabetic and antioxidant actions

**DOI:** 10.17179/excli2015-232

**Published:** 2015-08-04

**Authors:** Mina Hemmati, Elham Zohoori, Omid Mehrpour, Mehdi Karamian, Somaye Asghari, Asghar Zarban, Roya Nasouti

**Affiliations:** 1 Atherosclerosis and Coronary Artery Research Center, Birjand University of Medical Sciences, Birjand, Iran; 2 Department of Biochemistry, Faculty of Medicine, Birjand University of Medical Sciences, Birjand, Iran; 3 Cellular and Molecular Research Center, Birjand University of Medical Sciences, Birjand, Iran

**Keywords:** Atherogenic dyslipidemia, adiponectin, antioxidant, diabetes, medicinal plants

## Abstract

Atherogenic dyslipidemia, characterized by an increased level of lipoprotein (a) and a decreased level of adiponectin, is a major risk factor for cardiovascular diseases in diabetic patients*.* To reduce cardiovascular risk in diabetic patients, use of agents with antidiabetic and anti-atherogenic potential is required. Using an animal model of diabetes, we investigated the antiatherogenic potential of extracts of three medicinal plants: jujube, barberry, and saffron. For this, serum level of fasting blood glucose, lipid profile, malondialdehyde, total antioxidant capacity, adiponectin and lipoprotein (a) in diabetic control and extract treated groups were measured. Statistical analysis of measurements showed that serum levels of fasting blood glucose, triglyceride, and VLDL decreased significantly (*P < 0.05*) in all treated groups. Treatment with all extracts reduced lipid peroxidation and increased antioxidant capacity of the experimental diabetic groups. Serum adiponectin levels increased in all treated groups, whereas lipoprotein (a) levels decreased, most markedly when treated with jujube extract. Jujube, saffron, and barberry extracts are beneficial in ameliorating oxidative stress and atherogenic risk of diabetic rats. This highlights the benefits of further investigating the cardio-protective potential of medicinal plant extracts and evaluating their usefulness as cardio protective agents in clinical practice.

## Introduction

The increasing incidence of diabetes mellitus (DM) is a major global public health concern (Shane-McWhorter, 2005[32]). Diabetes requires long-term medical care for glycemic control; decreases the quality of life because of complications (such as retinopathy, neuropathy, and nephropathy); and leads to a large increase in medical expenditure (Chen et al., 2013[8]). In addition, diabetes increases the risk of life-threatening diseases, including cardiovascular diseases (CVDs) and cancer (Seshasai et al., 2011[31]). The prevention of diabetes is therefore an issue of high priority. There is considerable evidence that hyperglycemia-mediated oxidative stress is the major cause of complications of DM (Davi et al., 2005[11]; Pari and Latha, 2005[27]) and lipid peroxide-mediated tissue damage is observed in the development of type I and type II DM (Feillet-Coudray et al., 1999[13]). Dyslipidemia is a major CVD risk factor in DM. The characteristic features of diabetic dyslipidemia are high plasma triglyceride levels, low HDL cholesterol levels, and increased concentrations of small dense LDL-cholesterol particles (Cohn and Sernyak, 2006[10]). Several studies report high plasma levels of lipoprotein (a) [Lp(a)] in diabetic patients (Levitsky et al., 1991[21]). Lp(a) consists of a carbohydrate-rich protein named apo(a) linked by a single disulfide bond to apoB of an LDL-like lipoprotein. Like LDL, Lp(a) is thought to be atherogenic. Because of the structural homology of apo(a) to plasminogen, it is also thought to have thrombogenic properties. The possibility of proatherogenic and prothrombotic properties has led to a surge in research on the role of Lp(a) in atherosclerosis (Purnell et al., 1995[28]). The atherogenic index of plasma (AIP), defined as the logarithm (log) of the plasma concentration ratio of triglycerides to high-density lipoprotein (HDL) cholesterol, was recently proposed as a predictive marker for plasma atherogenicity, and is positively correlated with the risk of CVD (Geohas et al., 2007[15]). Adipose tissue is considered as an important endocrine organ that secretes many biologically active substances, collectively known as adipocytokines (Satoh et al., 2004[30]). The major adipocytokine, adiponectin, is thought to play an important role in the regulation of cardiovascular and metabolic homeostasis. Adiponectin increases tissue fat oxidation, leading to reduced levels of fatty acids and tissue triglyceride content, thus increasing insulin sensitivity (Satoh et al., 2004[30]). Paradoxically, diabetes patients have decreased plasma adiponectin concentrations (Ashiuchi et al., 2002[2]), suggesting that hypoadiponectinemia is involved in the pathophysiology of DM. Another key element in development and progression of DM is oxidative stress, and use of antioxidants such as vitamins E and C improves the action of insulin in diabetic patients (Ceriello, 2000[7]; Maritim et al., 2003[23]). Accordingly, dietary supplementation with nutrients rich in antioxidants could ameliorate hyperglycemia-mediated stress in DM. Traditional antidiabetic plants might provide new oral hypoglycemic compounds which may counter the high cost and poor availability of the current medicines.* B. vulgaris,* *C. sativus, *and* Z. jujuba* have antidiabetic and antioxidant effects on diabetic patient (Farkhondeh and Samarghandian, 2014[12]; Kaleem et al., 2014[20]; Meliani et al. 2011[24]). There is a direct correlation between antioxidant activity and phenolic content of *Z. jujuba* (Li et al, 2005[22]). *B. vulgaris* has a high antioxidant capacity due to its high levels of total phenolic, flavonoid (such as anthocyanin), and other polyphenolic compounds (Özgen et al, 2012[26]). The methanolic extract of *Crocus sativus *and its components such as safranal, crocin, etc. are reported to have radical scavenging activity (Bhargava, 2011[6]). To better understand the action of *B. vulgaris,* *C. sativus, *and* Z. jujuba* in diabetes, and also to compare between effectiveness of the plants in treatment of diabetes, we studied the effects of hydroalcoholic and aqueous extracts of these plants on atherogenicity and risk of CVD in experimental diabetes.

## Materials and Methods

### Animal experiments

Adult male Wistar-derived rats with a mass of 200 to 220 g, bred and raised at the Birjand University of Medical Sciences animal quarters, housed at five rats per cage, were fed a rat chow diet (Pars Dam Co, Tehran, Iran) and given water *ad libitum*. Diabetes was induced by intraperitoneal injection of streptozotocin (60 mg/kg body mass) two weeks before starting treatment. Two weeks after injection, animals with plasma glucose levels exceeding 16.6 mM were considered to be diabetic. We randomly grouped 65 diabetic rats as follows: Groups 1 to 6 comprised of diabetic rats receiving hydroalcoholic extracts of barberry, saffron, and jujube at doses of 25 and 100 mg/kg, respectively. Groups 7 to 12 received an equal dose of aqueous extracts of these plants. Extracts were administered orally for 21 days. Diabetic (group 13) and healthy (group 14) control rats received water instead of extract. At the end of the 3-week treatment, blood samples were collected and biochemical parameters were measured. Different concentrations of aqueous and alcoholic extracts of the plants were prepared on the basis of previous studies (Taati et al., 2011[34]; Abdel-Zaher et al. 2005[1]). Results of the similar studies led us to use the selected doses of the medicinal plants (Meliani et al., 2011[24]; Gulfraz et al., 2008[17]).

### Measurement of FBG and lipid profile, and assessment of the atherogenic index

Serum concentrations of FBG, triglyceride, total cholesterol, and HDL-C were measured by photometric methods using diagnostic kits (Pars Azmun, Iran). The atherogenic index of plasma (AIP), calculated as log [TG]/[HDL-C] is used as a significant predictor of atherosclerosis (Nwagha et al, 2010[25]).

### Measurement of Adiponectin and Lp(a)

Serum adiponectin and Lp(a) concentrations were estimated using specific enzyme-linked immunosorbent assays (Glory Sciences, Taiwan). For each group, assays were performed in duplicate. The experimental protocol was approved by the ethics committee of the Birjand University of Medical Sciences.

### Ferric Reducing Antioxidant Power (FRAP) Assay

Total serum antioxidant power was measured with the FRAP assay of Benzie and Strain (1996[5]). In brief, antioxidants in serum reduce ferric iron to its ferrous form at low pH, leading to the formation of a colored ferrous-tripyridyltriazine complex. Two ml of FRAP reagent (300 mM sodium acetate buffer pH 3.6, 10 mM 2,4,6-tris (2-pyridyl)-S-triazine (TPTZ) in HCl, and 20 mM FeCl3.6H2O, mixed in a proportion of 10:1:1 (v/v) was added to 50 µl of sample. After 15 min, the absorbance at 593 nm was read (Benzie and Strain, 1996[5]).

### Measurement of Thiobarbituric Acid Reactive Substances (TBARS)

Malondialdehyde (MDA) levels were measured using the Thiobarbituric acid reactive substances (TBARS) method (Basu et al, 2009[4]) in blood, collected from the heart at the end of treatment period. Plasma samples (300 µl) were added to 3 ml TBARS reagent (7.5 g trichloroacetic acid, 187 mg TBA, and 6.25 ml chloridric acid), the mixture was heated in boiling water bath for 20 min, and the absorbance at 532 nm was determined.

### Statistical analysis

Data was analyzed using One-Way ANOVA with SPSS version 16 software (SPSS Inc., Chicago, IL, USA). The statistical significance of differences in mean levels of FBS, Lp(a), etc. between the control and treated groups was evaluated using Student’s t-test. P-values of 0.05 or less were considered significant. Graphs were drawn with GraphPad Prism version 5 (GraphPad Software Inc., La Jolla, CA, USA).

## Results

Compared with the diabetic control group, rats treated for 21 days with aqueous or hydroalcoholic extracts of* B. vulgaris*,* C. sativus*, and* Z. jujuba* had significantly reduced serum levels of FBG and triglyceride (p < 0.05) (Tables 1(Tab. 1) and 2(Tab. 2)). Oral administration of these extracts also significantly improved lipid profile of diabetic rats. Consumption of *B. vulgaris*,* C. sativus*, and* Z. jujuba* reduced total cholesterol levels to those of the normoglycemic control group (Tables 1(Tab. 1) and 2(Tab. 2)), and in contrast only Z*. jujuba* extracts at the two specified doses could increase HDL-C levels. Results represented in Tables 1 and 2, indicate hypolipidemic effects of the plants used in the present study. *B. vulgaris*,* C. sativus*, and* Z. jujuba* could improve increased level of triglycerides in diabetic rats. Compared to the nondiabetic control group, diabetic control rats had significantly increased serum Lp(a) levels (p < 0.05) (Table 1(Tab. 1)). Treatment with the plant extracts reduced their Lp(a) levels to those of the nondiabetic control group. The result presented in Table 1(Tab. 1) and 2(Tab. 2) show that diabetic control group had lower levels of adiponectin than normoglycemic group. Treatment with saffron, jujube, and barberry extracts increased their adiponectin levels. The AIP of diabetic rats decreased significantly following administration of saffron, jujube and barberry extracts (Tables 1(Tab. 1) and 2(Tab. 2)). In Tables 1(Tab. 1) and 2(Tab. 2), figures bearing different superscripts (a and b) are significantly different at p < 0 .05 (one way ANOVA and Duncan test).

**Figure 1:**
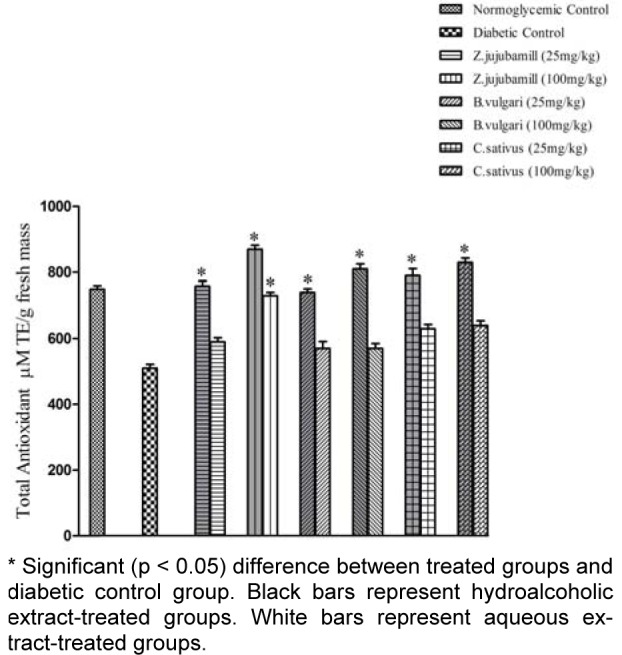
The effect of hydroalcoholic and aqueous extract of barberry, saffron and jujube on antioxidant capacity (Mean ± SD).

**Figure 2:**
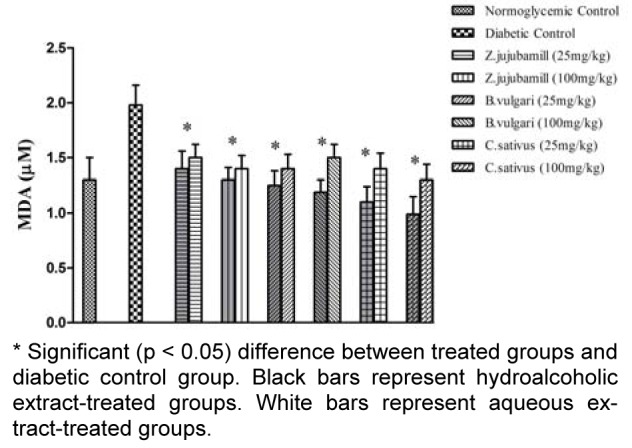
The effect of hydroalcoholic and aqueous extract of barberry, saffron and jujube on malondialdehyde (Mean ± SD).

The plant extracts ameliorated hyperglycemia-mediated oxidative stress in diabetes and improved the antioxidant capacity of the experimental groups compared to diabetic control group (Figures 1(Fig. 1) and 2(Fig. 2)). As shown in Figure 1(Fig. 1), the hydroalcoholic extracts increased antioxidant capacity more effectively than the aqueous extracts. This effect was more notable when using the jujube extract than with the other extracts. As shown in Figure 2(Fig. 2), the medicinal plant extracts reduced lipid peroxidation. The saffron extracts were more effective than the other plant extracts, and the hydroalcoholic extract was more effective than the aqueous extract.

## Discussion

It is well known that the incidence of CVD in patients with diabetes is high. Although CVD pathogenesis in diabetes is multifactorial, dyslipidemia is a powerful risk factor (Suryawanshi et al., 2006[33]). In addition, oxidative stress has a key role in this process (Hambali et al., 2011[19]). In the present study antiatherogenic and antioxidant effects of three medicinal plants *B. vulgaris*,* C. sativus, *and *Z. jujuba* were compared. Our results show that saffron, jujube, and barberry extracts may have antiatherogenic effects in diabetic rat models, which is likely to be related to the antioxidant capacities of the extracts. We found that treating diabetic rats with aqueous and hydroalcoholic extracts of* B. vulgaris*,* C. sativus, *and *Z. jujuba* effectively decreased their elevated FBS levels. The hypoglycemic effects of the medicinal plants were previously reported (Meliani et al., 2011[24]). Oral administration of hydroalcoholic extracts of *C. sativus*, *B. vulgaris,* and *Z. jujuba* significantly (*P *< 0.05) increased the serum adiponectin levels of diabetic rats. Considering the results of similar studies, we may conclude that adiponectin has a reverse relationship with glucose, triglyceride, VLDL, and cholesterol, and a direct relationship with HDL-C (Qiao et al., 2008[29]). Given the role of adiponectin in glucose and lipid metabolism, it seems that the hypoglycemic and hypolipidemic effects of the plants could be affected through changes in adiponectin levels. The AIPs of the different groups indicate that saffron, jujube and barberry extracts have anti-atherogenic effects (Table 1(Tab. 1), 2(Tab. 2)). Concentrations of Lp(a) were higher in the diabetic control group than in the nondiabetic control group (Table 1(Tab. 1)). Elevated levels of Lp(a) in type I DM were found in other studies (Haffner, 1993[18]; Giacco and Brownlee, 2010[16]). Serum Lp(a) levels significantly decreased in all treated groups (Tables 1(Tab. 1) and 2(Tab. 2)). These medicinal plant extracts may therefore reduce the high risk of cardiovascular complications in experimental diabetes. Use of antioxidant-rich nutrients to reduce atherogenic modifications has been studied previously (Aviram et al., 2000[3]). Our measurement of antioxidant capacity in diabetic rats revealed high glucose- mediated oxidative stress, which was relieved by oral consumption of jujube, saffron, and barberry extracts. Among the different plant extracts used in our study, jujube had a higher antioxidant capacity. It appears that use of natural sources of antioxidants such as barberry, saffron, and especially jujube, is useful in treatment of diabetes and associated CVD. It was suggested that the possible antioxidant activities of extracts are due to the presence of tannins, carotenes, and flavonoids in some *Ziziphus* species (Gao et al., 2012[14]; Taati et al., 2011[34]) and a direct correlation was found between the antioxidant activity and phenolic contents (Li et al., 2005[22]). Adiponectin as an antioxidant is inversely correlated with oxidative stress, inflammation, and chronic diseases such as diabetes (Chen et al., 2012[8]). Dyslipidemia is a common feature of DM. Our study supports the finding that the diabetic dyslipidemia comprising of elevated total cholesterol, TG, VLDL, Lp(a), and low HDL-C and adiponectin levels persists in diabetes. Lp(a) may be considered as an important independent risk marker to identify diabetes patients who may develop CVD. In this study, we found that jujube, saffron, and barberry extracts especially hydroalcoholic extract could reduce risk of CVD by improving the lipid profile, AIP, and Lp(a) levels. TG and HDL are the parameters that affect AIP. In the present study, herbal medicine consumption improved lipid profile, also AIP decreased to normoglycemic group. According to the role of Lp(a) in atherogenicity, and considering that consumption of the extracts especially jujube reduced Lp(a), we can expect reduced level of AIP in treatment groups. A more accurate explanation will obtain from consumption of the extracts by normoglycemic rats and investigation of changes in lipid profile and AIP of the experimental groups. According to the results of the FRAP assay, oral administration of the extracts increased total antioxidant capacity and AIP in treatment groups could be in associate with total antioxidant capacity. These beneficial effects especially for saffron and jujube could be taken into consideration when designing new drugs. Based on our data, adding jujube and saffron extracts to diets for diabetic patients may help to improve control of diabetic complications. Further experimental and clinical investigation is needed to determine the effective dosage of the plant extracts in clinical practice.

## Acknowledgements

This investigation was supported by Grant No. 711 from the office of Vice Chancellor for Research, Birjand University of Medical Sciences.

## Conflict of interest

The authors declare that they have no conflict interests.

## Figures and Tables

**Table 1:**
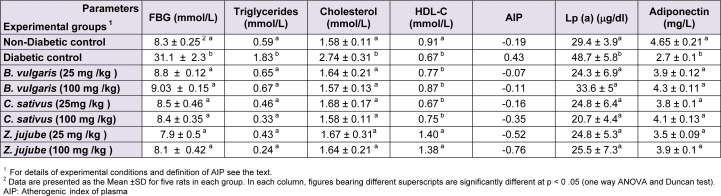
The effects of hydroalcoholic extracts of *B. Vulgaris, C. Sativus and Z. Jujuba* on lipid profile and AIP in diabetic groups

**Table 2:**
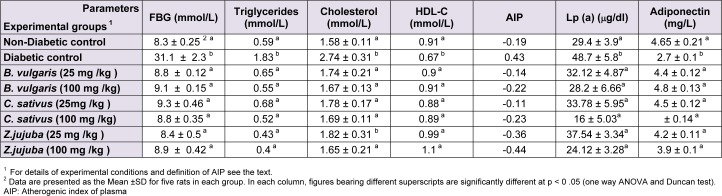
The effects of aqueous extracts of *B. Vulgaris, C. Sativus and Z. Jujuba* on lipid profile and AIP in diabetic groups
